# Clinical characteristics of Kawasaki disease with pulmonary radiographic abnormalities and its impact on the incidence of coronary artery lesions: a randomized retrospective cohort study

**DOI:** 10.3389/fped.2025.1506735

**Published:** 2025-02-06

**Authors:** Chenchen Liu, Xing Rong, Huixian Qiu, Jinhui Zhou, Yufei Chen, Xuhong Huang, Maoping Chu, Zhenquan Wang

**Affiliations:** Children’s Heart Center, The Second Affiliated Hospital and Yuying Children’s Hospital, Institute of Cardiovascular Development and Translational Medicine, Wenzhou Medical University, Zhejiang, China

**Keywords:** Kawasaki disease, pneumonia-like changes, pulmonary complications, coronary artery lesions, logistic

## Abstract

**Objective:**

The aim of this study was to investigate the characteristics of Kawasaki disease (KD) in patients demonstrating pneumonia-like changes and pulmonary complications, as well as the subsequent impact on coronary artery lesions, by comparing them with those of KD patients with normal pulmonary imaging.

**Method:**

From January 1, 2013 to October 1, 2022, this study included paediatric patients diagnosed with KD who were registered in the KD database at Yuying Children's Hospital affiliated with Wenzhou Medical University. Patients were divided into three distinct groups based on the presence and severity of abnormalities observed via lung imaging. We first compared the demographic and clinical characteristics across these groups. The imaging characteristics of KD patients with pneumonia-like changes and pulmonary complications were identified via chest radiography (x-ray) and chest computerized tomography (CT). Logistic regression models and stratified analyses were employed to further identify factors influencing coronary artery lesions (CALs).

**Results:**

Among the 2,686 KD children admitted to our centre in recent years, 115 presented with pneumonia-like changes, 366 presented with pulmonary complications, and 495 presented with no evident abnormalities on chest radiographs. In KD patients with pneumonia-like changes, there were significant elevations in inflammatory markers including the C-reactive protein (CRP) (*P* = 0.011), white blood cell (WBC) (*P* = 0.027), NT-proBNP (*P* = 0.007), and D-dimer (D-D) (*P* = 0.002) levels. Imaging studies have frequently revealed bilateral lung infections, predominantly in the mid-lower lung fields. Bronchitis-related changes were the most common manifestation of pulmonary complications in KD patients. A significant difference was observed in the incidence of CALs among patients with pneumonia-like changes. After adjusting for confounding variables, patients with pneumonia-like changes had a greater likelihood of developing CALs, with an adjusted odds ratio (OR) of 1.94 [95% confidence interval (CI): 1.21, 3.11]. Similar findings were obtained through stratification and sensitivity analyses.

**Conclusion:**

Patients diagnosed with KD who develop pneumonia-like changes and related pulmonary complications can be identified based on their clinical manifestations and imaging characteristics. Moreover, patients with KD and pneumonia-like changes had a significantly increased risk of developing CALs.

## Introduction

Kawasaki disease (KD) is a systemic multisystem vasculitis that primarily affects children under the age of 5 (1, 2). Coronary artery lesions (CALs) are the most common complication of KD and include coronary artery dilatations and coronary artery aneurysms (CAAs) (3–5). Most CALs that occur in the acute phase gradually resolve over time (6), however, the conditions of certain children with CALs persist or progress, leading to stenosis or obstruction and even acute myocardial infarction (6, 7). Therefore, KD is considered a major cause of acquired heart disease in children in developed countries and a potential risk factor for ischaemic heart disease in adulthood (8, 9). Given its nature as a vasculitis, KD can impact various bodily systems, such as the respiratory, gastrointestinal, central nervous, and genitourinary systems (10). The associated clinical manifestations include cough, shortness of breath, vomiting, abdominal pain, diarrhoea, gallbladder effusion, jaundice, urethritis, arthritis, and aseptic meningitis (11–16).

The occurrence rate of pulmonary involvement varies with ethnicity. A case series conducted in Japan revealed that 14.7% of patients demonstrated pulmonary changes on the chest x-ray (17). Conversely, a study of 250 patients in Italy reported no cases of pulmonary involvement (18). A retrospective cohort study reported an incidence of 1.83% for pulmonary manifestations of KD (19). However, KDs associated with pneumonia-like changes and pulmonary complications may present distinct characteristics. For example, a study reported a neonate with atypical KD and severe pneumonia requiring mechanical ventilation (20). Previous studies reported that KD combined with pulmonary abnormalities does not respond to antibiotics.

In KD, the absence of characteristic clinical symptoms and abnormal laboratory indicators, coupled with atypical lung imaging findings, complicates the recognition of the condition. This often results in a delayed diagnosis and subsequent postponement of intravenous immunoglobulin (IVIG) therapy (21). Therefore, early identification and diagnosis are crucial for promptly determining the underlying cause, facilitating a timely treatment and preventing the development of coronary artery disease (22). Studies have shown that a subset of atypical KD patients initially present with pulmonary symptoms, such as pneumonia, pleural effusion, and pneumothorax. Research suggests that in paediatric patients exhibiting persistent fever despite appropriate antibiotic treatment, pneumonia may serve as an important indicator for diagnosing KD (19).

This study aimed to analyse the clinical characteristics of patients diagnosed with KD who presented pulmonary imaging abnormalities. Additionally, we investigated the potential correlation between these imaging abnormalities and the presence of CALs.

## Methods

We enrolled a total of 2,686 patients with KD who were hospitalized from January 1, 2013 to October 1, 2022 at the Yuying Children's Hospital of Wenzhou Medical University. The diagnosis of complete KD was based on the criteria established by the American Heart Association (AHA) (23) which indicates a fever lasting for at least 5 days, in conjunction with four of the following five major clinical criteria: rash, bilateral non-exudative conjunctivitis, inflammation of the oral mucosa, cervical lymphadenopathy, and extremity changes. The diagnosis of incomplete KD adhered to the criteria outlined by the AHA (23).

Pneumonia-like changes are identified by radiological evidence of abnormal inflammatory density within the lung parenchyma, according to the diagnostic criteria (24–29). Pulmonary complications are characterized by a range of alterations and non-specific manifestations, including common bronchitis, turbidity surrounding bronchial vessels, the formation of pulmonary nodules or dead spaces, bronchial hypertrophy or necrosis, and stenosis. These conditions may be accompanied by pulmonary ground-glass opacities, pneumothorax, pleural effusion, and pulmonary microinfarction, among other symptoms (30, 31). Consequently, imaging results of the lung were reviewed by a KD specialist to screen pulmonary abnormalities.

Coronary artery anomalies (echocardiography) were classified using a scheme based exclusively on *Z* scores as follows: (1) No involvement: Always <2 mm. (2) Dilation: 2 mm to <2.5 mm. (3) Small aneurysm: ≥2.5 mm to <5 mm. (4) Medium aneurysm: ≥5 mm to <10 mm, and absolute dimension <8 mm (5). Large or giant aneurysm: ≥10 mm, or absolute dimension ≥8 mm (23). Given patient compliance, echocardiography was utilized to monitor patients from the onset of KD through 30 days post-diagnosis.

IVIG resistance: Persistent or recrudescent fever at least 36 h and less than 7 days after completion of initial IVIG infusion (23).

Treatment regimen: During the acute phase, all patients received aspirin at a daily dosage ranging from 30 to 50 mg/kg. Depending on the total immunoglobulin dose and body weight of the child, the regimen was divided into two types: standard regimen (2 g/kg, single dose intravenously) and non-standard regimen (for example, 1 g/kg/days for two consecutive days) (32–34).

Duration of IVIG treatment: IVIG treatment received more than 10 days after onset of illness was defined as delayed IVIG treatment, otherwise, it was considered non-delayed IVIG treatment.

Clinical findings, laboratory parameters, and radiological data were systematically documented. Prior to the administration of IVIG, laboratory test results were collected upon patient admission. These results included measurements of the white blood cell count (WBC), C-reactive protein (CRP), albumin (ALB), alanine aminotransferase (ALT), platelet count (PLT), N-terminal pro-B-type natriuretic peptide (NT-proBNP), and D-dimer (D-D). Additionally, other laboratory indicators potentially indicative of specific or confounding factors associated with CALs were incorporated as covariates in the analysis. Radiological assessments included chest radiography or chest computerized tomography when necessary, along with echocardiography for the evaluation of CALs.

**Figure 1:**
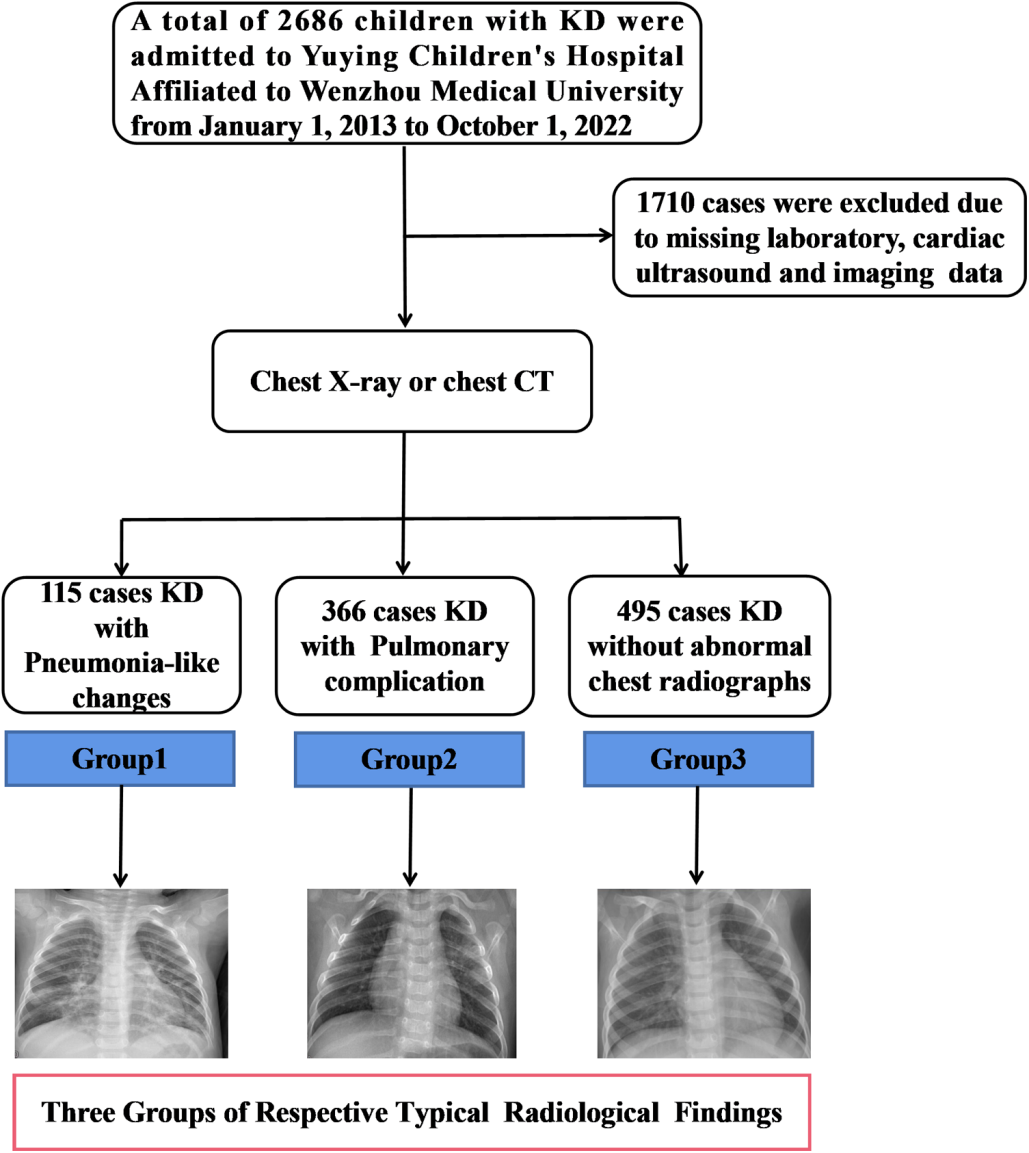
Patients flow chart. Flow chart showing the demographic and clinical information of all study participants. From 1 January 2013 to 1 October 2022, 2,686 children in our Kawasaki disease database were enrolled.

Patients who were diagnosed with KD and who also presented with COVID-19 or multisystem inflammatory syndrome (MIS-C) were excluded from the study. Initially, 2,686 patients were registered in this study. Among them, 884 cases were excluded owing to the absence of pulmonary imaging evidence. Among the remaining 2,102 patients, an additional 584 patients were excluded because they did not undergo echocardiographic examinations at our hospital within one month of the onset of illness. Additionally, 242 patients were excluded because critical laboratory data were missing, including CRP, WBC, and ALB levels. The final cohort of 481 patients demonstrating abnormal pulmonary imaging was divided into two groups based on the presence of infiltrative shadows or interstitial changes identified via chest x-ray or CT. Group 1 included 115 patients diagnosed with pneumonia-like changes, whereas Group 2 included 366 patients with various pulmonary complications. Furthermore, a control group (Group 3) included 495 patients admitted during the same timeframe who exhibited no significant abnormalities on the chest x-ray or CT. All patients across these groups received IVIG therapy (Figure 1).

### Statistical methods

In the first step, the demographic and clinical characteristics of the three groups were compared. Additionally, multiple logistic regression models were used to assess the differences in the relevant clinical indicators between Group 1 and Groups 2 and 3, as well as to assess the impact of pulmonary radiographic abnormalities and other factors on CALs. Model 1 was adjusted for age (in months) and sex alone. Model 2 was adjusted for age (in months), sex, type of KD, IVIG treatment outcome, treatment delay, and treatment regimen. Model 3 was further adjusted for laboratory indicators. Stratified analyses were also conducted to further validate the impact on CALs in the different subgroups of patients.

Categorical variables were analysed via the chi-square test. Continuous variables were analysed with either a *t*-test or a non-parametric test, depending on the results of the normality test. A *p*-value of less than 0.05 was considered statistically significant. All the statistical analyses were performed via IBM SPSS (version 26).

Finally, we conducted a quantitative assessment of chest radiographs and chest computed tomography scans in two cohorts of KD patients exhibiting pulmonary imaging abnormalities. Subsequently, we analysed the specific radiological changes and identified the predilection sites for these abnormalities.

## Results

KD patients with combined pneumonia-like changes and pulmonary complications exhibit distinct onset characteristics.

Compared with the other two groups, the group of KD patients with pneumonia-like changes had a significantly greater proportion of CALs (*P* < 0.001), higher rate of antibiotic treatment prior to hospitalization (*P* = 0.002), and greater prevalence of the comorbidities of cough (*P* < 0.001). Upon admission, all patients presented with clinical symptoms, including fever, a relatively severe cough, and a rapid respiratory rate, with 80% of patients exhibiting a rate exceeding 30 breaths per minute. Auscultation revealed that wet rales were the most prevalent lung sound, occurring in 46.09% of the cases, followed by wheezing at 23.48%, whereas dry rales were less common, occurring in only 3.49% of the patients (refer to Table 1). These patients also demonstrated significantly longer durations of hospitalization (*P* < 0.001) and elevated levels of CRP (*P* = 0.011*)*, WBC (*P* = 0.027), PLT (*P* = 0.009), ALB (*P* = 0.004), NT-proBNP *(P* = 0.007), and D-D (*P* = 0.002). Additionally, there was a higher rate of resistance to IVIG (*P* = 0.003). Conversely, no significant differences were found in age, type of KD, or other factors. Furthermore, the differences in these indicators between Group 2 and the control group were not statistically significant (Table 2).

**Table 1:** Clinical symptoms and imaging findings of Kawasaki disease with pneumonia-like changes.

Clinical symptoms	*N*	Percentage/%	
Fever	115	100	
Cough	28	24.35	
Moist rosette in the lungs	53	46.09	
Dry rumbling in the lungs	4	3.49	
Wheezings	27	23.48	
Pathological changes			
Regions	Both lungs	78	67.83
	Left lung	21	18.26
	Right lung	16	13.91
	Upper lobe of the left lung	6	5.22
	Lower lobe of the left lung	15	13.04
	Upper lobe of the right lung	3	2.61
	Lower lobe of the right lung	12	10.43
Imaging features	Small patchy shadow	80	69.57
	patchy shadow	35	30.43
	Partial consolidation	2	1.74
	Merge pleural effusion	2	1.74

**Table 2:** Basic characteristics of all Kawasaki disease patients.

Characteristics	Pneumonia-like changes (*N* = 115)	Pulmonary complications (*N* = 366)	Control group (*N* = 495)	*P*-value
Age (months)	20.5 (8.6–37.4)	20.5 (11.6–38.0)	26 (13.9–41.8)	0.099
Male, *n* (%)	32 (27.8)	139 (38.0)	199 (40.2)	0.048
Cough, *n* (%)	28 (24.3)	51 (14.0)	16 (3.2)	<0.001
Digestive symptoms, *n* (%)	5.0 (4.3)	21 (5.7)	19 (3.8)	0.418
Extremity changes, *n* (%)	81 (70.4)	215 (58.9)	286 (57.8)	0.042
Conjunctival congestion, *n* (%)	107 (93.0)	333 (91.0)	434 (87.7)	0.125
Rash, *n* (%)	89 (77.4)	265 (72.6)	361 (73.1)	0.582
Cervical lymphadenopathy, *n* (%)	55 (48.2)	200 (54.9)	282 (57.2)	0.219
Inflammation of oral mucosa, *n* (%)	107 (93.0)	333 (91.2)	460 (92.9)	0.622
Hosdays (days)	8.0 (6.39–11.0)	7.0 (6.0–9.0)	7.0 (6.0–9.0)	<0.001
Incomplete Kawasaki disease, *n* (%)	18 (16.8)	65 (18.7)	93 (18.8)	0.890
CRP (mg/L)	86.6 (56.7–140)	75.2 (46.3–116.3)	74.8 (39.8–110.2)	0.011
Albumin (g/L)	39.1 (35.8–41.8)	40.8 (37.8–43.4)	40.9 (38.4–43.7)	0.004
CRP/ALB ratio	2.25 (1.25–3.86)	1.80 (1.04–3.00)	1.78 (0.95–2.73)	0.005
WBC (×10^9^/L)	15.5 (12.5–20.1)	14.1 (11.1–17.2)	14.9 (11.8–18.7)	0.027
PLT >450 × 10^9^/L, *n* (%)	35 (30.4)	63 (17.2)	100 (20.2)	0.009
ALT/AST>0.9, *n* (%)	58 (59.8)	138 (44.5)	172 (40.8)	0.003
NT-proBNP (pg/ml)	1,010 (371–3,220)	738 (249–2,250)	602 (263–1,910)	0.007
D-dimer (μg/ml)	1.85 (1.03–3.18)	1.38 (0.92–2.40)	1.34 (0.86–2.15)	0.002
FIB (g/L)	6.28 (5.16–7.33）	6.17 (5.34–7.09)	6.29 (5.48–7.35)	0.471
CALs, *n* (%)	65 (56.5)	143 (39.1)	126 (25.5)	<0.001
IVIG resistance, *n* (%)	10 (8.7)	28 (7.7)	15 (3.0)	0.003
Antibiotic, *n* (%)	103 (89.6)	273 (75.0)	367 (74.4)	0.002

Quantitative data were expressed as mean ± SD and compared with the *t*-test if normally distributed, otherwise expressed as median (inter-quartile range) and compared with the rank-sum test, and qualitative data were expressed as frequency (%) and were compared with the Chi-square test or the Fisher exact test as appropriate.

Kawasaki disease, Kawasaki disease; IVIG, intravenous immunoglobulin; CRP, C-reactive protein.

Level; WBC, white blood cell count; PLT, platelet count; ALB, albumin; ALT, alanine aminotransferase; FIB, fibrinogen; CALs, coronary artery lesions.

Independent impact of combined pneumonia-like changes and pulmonary complications on CALs.

In Table 3, the lung conditions of the three groups were presented as independent variables. To further assess the relationship between pneumonia-like changes and CALs in children with KD, we conducted a multivariate analysis with CALs as the outcome variable. The analysis incorporated various indicators, including the presence of pneumonia-like changes, sex, age of the child, incomplete KD, standard treatment, laboratory indices, IVIG resistance, delayed diagnosis, and treatment. After adjusting the potential confounders, the results indicated a greater likelihood of CALs in the groups presenting pneumonia-like changes and pulmonary complications. However, the impact of pulmonary complications on the incidence of CALs was not statistically significant.

**Table 3:** Independent effect of pneumonia-like changes and pulmonary complications on CALs in Kawasaki patients.

Exposure	OR (95% CI) *P*-value		
	Model 1	Model 2	Model 3
Pulmonary complications (*n* = 366)	1 (reference)	1 (reference)	1 (reference)
Pneumonia-like changes (*n* = 115)	2.08 (1.35, 3.21)	1.97 (1.25, 3.11)	1.94 (1.21, 3.11)
	0.001	0.003	0.006
Control group (*n* = 495)	1 (reference)	1 (reference)	1 (reference)
Pulmonary complications (*n* = 366)	1.78 (1.33, 2.39)	1.74 (1.27, 2.36)	1.68 (1.22, 2.31)
	<0.001	<0.001	0.001
Control group (*n* = 495)	1 (reference)	1 (reference)	1 (reference)
Pneumonia-like changes (*n* = 115)	1.92 (1.55, 2.37)	1.86 (1.48, 2.34)	1.77 (1.40, 2.24)
	<0.001	<0.001	<0.001

CALs, coronary artery lesions; IVIG, intravenous immunoglobulin; OR, odds ratio.

Model 1 adjusted for: age (months), and gender (male, female).

Model 2 adjusted for: model 1+ Kawasaki disease type (complete, incomplete), IVIG therapeutic effect (sensitive, resistance), treatment regimen (standard, non-standard), and time of IVIG treatment (delayed, non-delayed).

Model 3 adjusted for: model 2+ C-reactive protein level (mg/L), white blood cell count (10^9^/L,), platelet count (10^9^/L), and albumin (g/L).

### Stratification and sensitivity analysis

A stratified analysis demonstrated that despite adjusting the confounding factors, patients with pneumonia-like changes presented a greater risk for CALs. Despite the small sample size, the adjusted odds ratios remained statistically significant across the multiple subgroups (Table 4).

**Table 4:** Stratified analysis of the effect of pneumonia-like changes on CALs in Kawasaki patients.

Stratifification factor	*N*	All patients (*n* = 481)	
		OR (95% CI)^a^	*P*-value
Age (months)			
≤16	189	1.98 (0.99, 3.98)	0.053
>16	292	2.12 (1.23, 3.68)	0.007
Sex			
Male	310	2.32 (1.39, 3.87)	0.001
Female	171	1.48 (0.69, 3.21)	0.316
CRP (mg/L)			
≤70	209	1.97 (0.98, 3.95)	0.055
>70	272	1.94 (1.13, 3.33)	0.016
WBC (×10^9^/L)			
≤18	357	1.57 (0.93, 2.67)	0.092
>18	124	3.62 (1.68, 7,77)	0.001
PLT (×10^9^/L)			
≤400	335	1.55 (0.92, 2.62)	0.098
>400	146	3.26 (1.54, 6.91)	0.002
ALB (g/L)			
≤43	350	1.86 (1.15, 3.01)	0.011
>43	131	2.37 (0.95, 5.92)	0.059
CRP/ALB (ratio)			
≤0.8	273	1.94 (0.66, 5.66)	0.223
>0.8	208	2.03 (1.28, 3.23)	0.002
NT-proBNP (pg/ml)			
≤100	264	0.67 (0.06, 7.23)	0.738
>100	198	1.97 (1.27, 3.07)	0.002
D-dimer (mg/L)			
≤0.5	256	3.50 (0.43, 28.45)	0.232
>0.5	187	2.10 (1.33, 3.32)	0.001

CALs, coronary artery lesions; Kawasaki disease, Kawasaki disease; CRP, C-reactive protein; WBC, white blood cell; PLT, platelet count; ALB, albumin.

^a^ Adjusted for: age (months), gender (male, female), C-reactive protein level (≤70 mg/L, >70 mg/L), white blood cell count (≤18 × 10^9^/L, >18 × 10^9^/L), platelet count (≤400 × 10^9^/L, >400 × 10^9^/L), and alanine aminotransferase level (≤43 U/L, >43 U/L), CRP/ALB level (≤0.8, >0.8), NT-proBNP level (≤100 pg/ml, >100 pg/ml), D-dimer level (≤0.5 mg/L, >0.5 mg/L).

### Imaging findings

**Figure 2:**
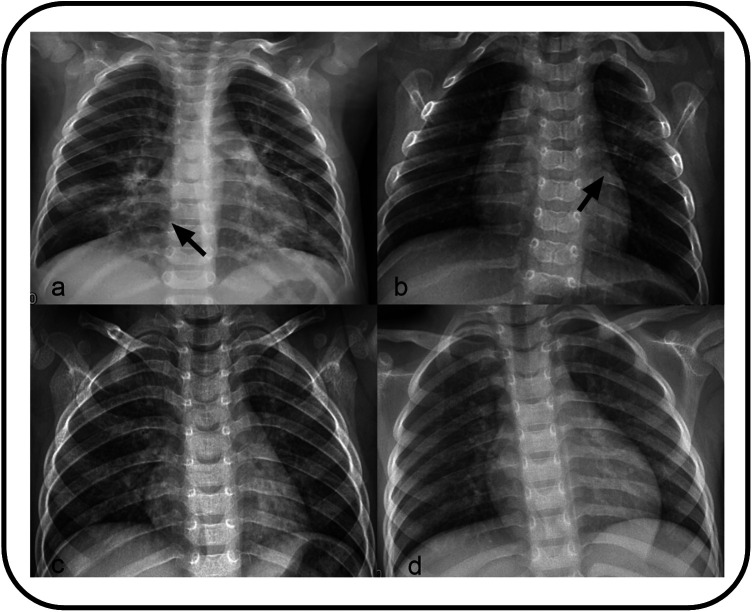
**(a)** Showing small patchy blurred shadows distributed along the lung texture in bilateral lungs, with uneven density. **(b)** Showing Patchy high-density shadows in the left upper lung. **(c)** Showing bronchitis. **(d)** Showing increased lung texture.

**Figure 3:**
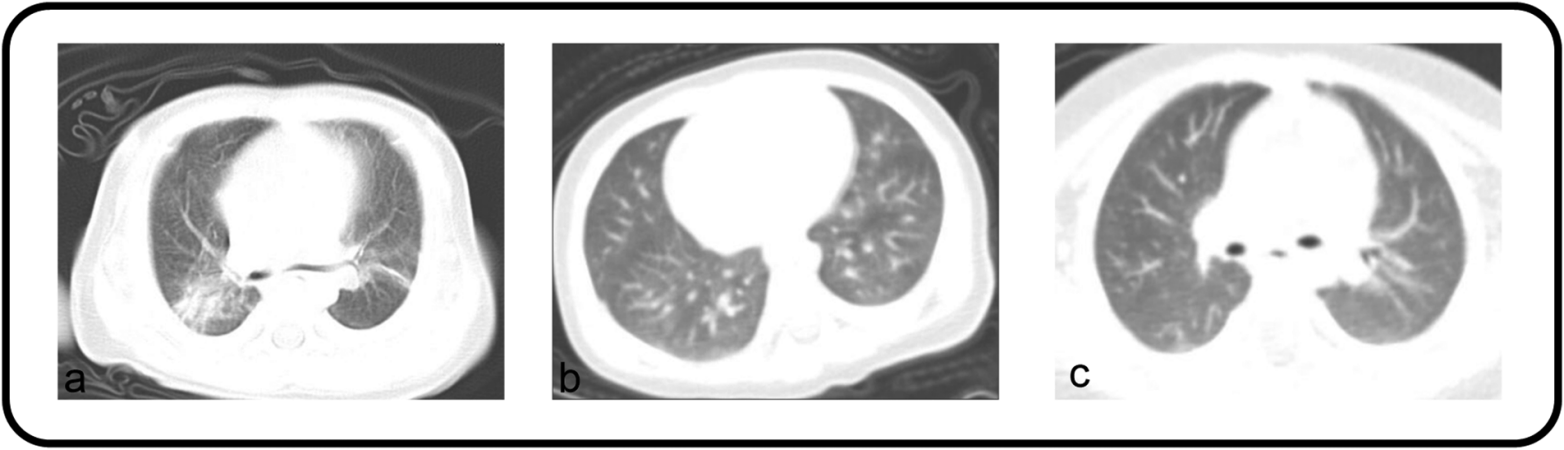
**(a,b)** Both patients’ chest CT showed bilateral lung infections. **(c)** Appearing as bronchitis on the chest CT scan.

All patients diagnosed with pneumonia-like changes underwent either chest x-ray or CT. The detailed imaging findings are summarized in Table 1. The majority of patients (67.83%) exhibited a bilateral distribution, among which 29 demonstrated the involvement of the lower lobes of both lungs. This was followed by the left lower lobe (13.04), right lower lobe (10.43), and right upper lobe (2.61) (Figure 2). Bilateral lung infections were observed on the chest CT of both patients (Figure 3). In this study, the majority of patients (69.57) demonstrated small, uneven shadows with a patchy density, whereas an increase in patchy density was observed in 35 patients (30.43). Pulmonary consolidation and pleural effusion were relatively rare, each occurring in only 2 patients (1.74), with pulmonary consolidation notably involving bilateral regions.

The specific imaging findings of 366 KD patients with pulmonary complications are summarized in Table 5. The most common finding was bronchitis, as illustrated in Figures 2, 3, which was observed in 63.39 of the cases. This was followed by an increase and blurred lung texture in both the left and right lungs, which was noted in 34.15 of the patients. Additionally, changes suggestive of bronchiolitis were identified in 7 patients, whereas pleural effusion was rare, occurring in only 2 patients.

**Table 5:** Imaging findings of Kawasaki disease with pulmonary complications.

Imaging features	*N*	Percentage/%
Bronchitis	232	63.39
Increased lung texture	125	34.15
Capillary bronchial-like changes	7	1.91
Pleural effusion	2	0.55

## Discussion

KD is considered a self-limiting disease; however, an increasing number of studies have supported the presence of vascular damage beyond the acute phase, which can lead to vascular stenosis, occlusion, and sudden acute death in adulthood (35). Unlike previous studies that primarily focused on patients with KD, this study specifically examined patients with KD accompanied by pulmonary radiographic abnormalities. Despite the administration of antibiotics to 103 KD patients with pneumonia-like changes prior to admission, fever persisted. The aim of this study was to identify distinguishing factors between concurrent pneumonia-like changes and pulmonary complications. Singh et al. reported that 1.3% of patients had pulmonary involvement and that 54.5% had pleural effusion (19). Ugi et al. reported a case in which an adult KD patient presented with lung involvement, especially bilateral massive pleural effusion (36). Pulmonary involvement in KD is extremely rare and can manifest as various types of changes, such as pneumonia-like changes, pulmonary nodules, pneumothorax, and pleural effusion.

Patients with pneumonia-like changes in the present study had a median white blood cell count of 15.5 × 10^9^/L, which was significantly greater than the median count of 14.1 × 10^9^/L observed in the group with pulmonary complications. The median CRP level in the group with pneumonia-like changes was 86.6 mg/L, which surpassed 75.2 mg/L reported in the cohort with non-pneumonia-like changes. We hypothesized that a WBC count exceeding 15 × 10^9^/L, combined with a CRP level above 70 mg/L, can assist in identifying KD patients with pneumonia-like changes. Elevated CRP levels may also be indicative of non-infectious diseases, such as vascular inflammation. Current epidemiological and pathogenic studies suggest that various infectious agents may be related to the occurrence of KD (37), with clinical manifestations of respiratory tract infections varying widely. For example, *Streptococcus pneumoniae*, *Mycoplasma pneumoniae* (MP), Chlamydia, adenovirus, enterovirus, parainfluenza virus, coronavirus, and Epstein–Barr virus have been implicated in KD (38). According to the superantigen hypothesis, excessive activation of T lymphocytes, coupled with the secretion of various cytokines following CD4^+^ T-cell activation, can promote the polyclonal activation, proliferation, and differentiation of B cells into plasma cells, leading to a significant increase in inflammatory factors and ultimately causing vasculitis (39, 40). Pulmonary involvement in KD patients may result from vasculitis, which is characterized by an increased vascular permeability and perivascular oedema (41, 42). During the acute phase of KD, VEGF levels significantly increase, leading to rashes and the extensive induration of the hands and feet (43). EGF, which is predominantly produced by vascular smooth muscle cells, increases the microvascular permeability, resulting in perivascular oedema (44). Limb changes in KD patients during the acute phase manifest as erythema and induration in the hands and feet, followed by desquamation approximately 2 weeks later; a greater frequency of these changes may be observed in KD patients with pneumonia-like changes.

Furthermore, our observations revealed that KD patients with pneumonia-like changes presented higher median of NT-proBNP and D-D levels than those with pulmonary complications. Research suggests that elevated NT-proBNP levels may result from local myocardial inflammation or ischaemic areas (45). Another mechanism may involve cytokines. Tumour necrosis factor (TNF)-α, which is present in the acute phase of KD, induces endothelial cells to express adhesion molecules for neutrophils and monocytes. TNF-α acts on endothelial cells and fibroblasts, promoting the production of various chemokines, thereby facilitating the migration of inflammatory cells to inflammatory sites and increasing cytokine production. These findings suggest that cytokines may play a role in promoting the secretion of acute-phase NT-proBNP in KD. We speculated that concurrent pneumonia-like changes may exacerbate the chemotactic effects of inflammatory factors, leading to a further increase in NT-proBNP levels. D-D is a degradation product of fibrin produced during fibrinolysis and is commonly used to indicate a hypercoagulable state. Our analysis suggested that when D-D exceeds 0.5 mg/L, it may help identify KD patients with pneumonia-like changes. Recent studies have identified D-Dr as a specific marker of the fibrinolytic system and an indicator of the inflammatory response and severe infection, demonstrating positive correlations with WBC, CRP, LDH, and ESR. Studies have also shown that D-D levels in children with systemic multisystem inflammatory syndrome are significantly elevated and correlate with the severity of the disease (46). During the acute phase of KD, patients exhibit an intrinsic elevation in platelet count. This study revealed a significant difference between the two groups when patients with a PLT over 450 × 10^9^/L were compared. Notably, many children with multisystem inflammatory syndrome present with elevated platelet levels, often above 400,000/μl upon admission (47). However, whether a comparable phenomenon is observed in infections caused by other pathogens or viruses remains unclear.

KD patients with pneumonia-like changes are at increased risk of CALs in the acute phase. Moreover, the presence of overlapping risk factors increases this probability. Umezawa et al. reported elevated serum CRP levels, a prolonged duration of positive CRP, and increased incidence of CALs in KD patients with abnormal chest radiographs (31). The development of CALs in KD patients is closely associated with inflammatory reactions (48). Pneumonia-like changes, as a complication of KD, represent an inflammatory reaction during the acute phase. Studies have shown that CRP levels are positively correlated with the size of CALs, serving as an independent factor influencing their persistence (49). A high WBC count is related to cardiac sequelae in KD patients (50). The ratio of CRP to albumin (CAR) has been identified as a novel predictive factor for the formation of CALs and IVIG resistance in KD patients (51). Compared with patients in the other groups, KD patients with pneumonia-like changes demonstrated significantly elevated CAR values (*P* = 0.005), indicating more severe inflammatory reactions and potentially a greater likelihood of developing CALs. He et al. described 34 KD infants who presented with lung involvement, which occurred at a higher rate than expected (52), and may be attributed to increased vascular permeability owing to vascular inflammation. Significantly elevated D-D and fibrinogen levels were observed in patients presenting with pneumonia-like changes, suggesting a hypercoagulable state that exacerbates the consequences of vasculitis. Additionally, a greater proportion of KD patients with pneumonia-like changes demonstrated delayed IVIG treatment, which further increased the risk of CALs (53, 54). These patients also had a higher rate of receiving non-standard treatment, which could further increase the likelihood of developing CALs. Singh et al. reported a high prevalence of CAAs in patients with pulmonary manifestations (27.3% of 11 KD cases), potentially due to delayed IVIG treatment (10). Studies have reported that MP infection may play a significant role in the development of coronary artery disease, suggesting a close association between MP and vascular changes. However, there is currently no evidence suggesting that MP infection exacerbates the condition of KD patients or increases the incidence of CAAs (55).

The pulmonary manifestations associated with KD may serve as indicators for evaluating disease severity. In KD patients presenting with pneumonia-like changes, the majority demonstrated bilateral pulmonary infections characterized by non-uniform density shadows predominantly located in the middle and lower lung fields. This pattern may indicate a more severe pulmonary form of KD. Furthermore, we hypothesized that this condition may be significantly correlated with the inflammatory responses observed in these patients. Bronchitis is the most common pulmonary complication in KD patients, demonstrating a high incidence of CALs and resistance to IVIG therapy. This phenomenon may be attributed to the relatively high prevalence of KD in children and the potential selection bias in patient inclusion. We hypothesized that the pulmonary complications associated with KD exhibited relatively mild imaging manifestations and that their impact on CALs and IVIG resistance was not significantly different from that observed in the control group. In KD patients accompanied with pulmonary complications, clinical manifestations may present atypically, and laboratory indices may not consistently exhibit the abnormalities. However, lung imaging plays a crucial role in facilitating an accurate diagnosis. A prompt diagnosis and appropriate treatment can potentially mitigate the incidence of CALs and resistance to IVIG.

Initially, pulmonary symptoms in patients with KD were predominantly managed with antibiotics. Our study included 103 patients who were diagnosed with KD and pneumonia-like changes, all of whom received antibiotic therapy during their hospital stay. Among these patients, 57 were administered cephalosporin antibiotics, which led to the complete resolution of all pulmonary symptoms during the follow-up period. However, in the majority of KD patients with pulmonary complications, the use of antibiotics may have disrupted the homeostasis of the intestinal microbiota, potentially impairing immune function and increasing the risk of secondary infections (56, 57). Changes in the gut microbiota may increase susceptibility to KD (58). Disturbances in the composition of the gut microbiota have been associated with systemic inflammation in KD patients (59). Early identification of these patients is crucial for avoiding the unnecessary use of antibiotics, thereby reducing antibiotic resistance, alleviating the global economic burden (60), and improving the cost-effectiveness of hospital treatment (61).

Recent management guidelines have not explored the influence of antibiotics on the incidence of CALs or the efficacy of IVIG in patients with KD. This study indicates that when pneumonia-like changes arise in the context of KD and antibiotic therapy becomes necessary, clinicians should perform a comprehensive evaluation of the underlying factors, including the severity of clinical symptoms, presence of significantly abnormal inflammatory markers, and occurrence of additional systemic complications. Unfortunately, throat swabs, sputum cultures, and respiratory virus tests were conducted on most patients; however, most of the results were negative. Among the limited positive findings, bacterial infections were either owing to colonization or specimen contamination, as these results normalized upon re-examination. In terms of the virus detection outcomes, 26 patients were identified as having Mycoplasma infections, as indicated by the presence of positive Mycoplasma IgM antibodies.

## Conclusion

Whether KD may be caused by pulmonary infection or the complications observed in the lungs are features of KD remains unclear. In summary, when assessing and diagnosing patients exhibiting pulmonary complications, which are characterized by an accelerated respiratory rate, atypical lung auscultation findings, elevated levels of CRP, WBC, NT-proBNP, and D-D, along with not responding effectively to standard anti-infection therapies and exhibiting a greater propensity for developing CALs, clinicians should maintain a high index of suspicion for pneumonia-like changes associated with KD. Prompt imaging assessments and the administration of IVIG therapy are critical for preventing the loss of the optimal treatment window (62, 63). Additionally, a comprehensive evaluation is necessary to ensure the rational use, modification, or escalation of antibiotic therapy. This study has several limitations. First, this was a retrospective study, and the high proportion of patients excluded owing to missing data may have led to selection bias; furthermore, other potential influencing factors, such as variations in treatment protocols among patients were not discussed. Additionally, as a single-centre study, the research was confined to a limited geographical area, potentially impacting the generalizability of the findings. Further investigations are essential to elucidate the differences between KD accompanied by pneumonia-like changes and pulmonary complications and to examine their relationship with CALs.

## Data Availability

The original contributions presented in the study are included in the article/Supplementary Material, further inquiries can be directed to the corresponding authors.
